# Kleine-Levin syndrome: clues to aetiology

**DOI:** 10.1007/s11325-017-1617-z

**Published:** 2018-03-12

**Authors:** Saad Mohammed AlShareef, Richard Mark Smith, Ahmed Salem BaHammam

**Affiliations:** 1 Department of Internal Medicine, College of Medicine, Al Imam Mohammad Ibn Saud Islamic University (IMSIU), Riyadh, Saudi Arabia; 2 Division of Medicine and Therapeutics, Ipswich Hospital NHS Trust and University of Suffolk, Heath Road, Ipswich, Suffolk, IP4 5PD UK; 3 The University Sleep Disorders Center and National Plan for Science and Technology, College of Medicine, King Saud University, Riyadh, Saudi Arabia

**Keywords:** Kleine-Levin syndrome, Recurrent hypersomnia, Aetiology, Autoimmunity, Functional neuroimaging

## Abstract

Kleine-Levin syndrome (KLS) is the commonest recurrent sleep disorder, with a prevalence of 1–2 per million population. Clear diagnostic criteria are now defined, but effective treatment remains elusive. The significant body of published literature allows consideration of possible aetiological mechanisms, an understanding of which could guide the development of therapeutic strategies. Functional imaging studies have been inconclusive; although diencephalic abnormalities are a common finding, no consistent pattern has emerged, and these studies have not revealed the mechanism(s) underlying the development of the abnormalities detected. An autoimmune aetiology is consistent with the available data. In this review, we argue that, in order to further our understanding of KLS, there needs to be a co-ordinated international effort to standardise approaches to functional imaging studies, genetic analyses that specifically address the possibility of an autoimmune aetiology, and clinical trials of immunosuppressive therapies.

## Introduction

Kleine-Levin syndrome (KLS) is the commonest recurrent hypersomnia, with a prevalence of 1–2 per million [1]. Despite significant advances in definition of the clinical syndrome, an effective treatment for KLS remains elusive. A better understanding of the aetiology of KLS will allow the proposal of therapeutic strategies.

Psychological theories of KLS were considered throughout the 1970s but dismissed in favour of ‘physical’ causes. Subsequent neuroanatomical, neurophysiological, biochemical, and more recently functional neuroimaging studies have added to our understanding. However, these imaging studies do not define the pathological processes causing the abnormalities detected. Possible causes include a purely psychological aetiology, physical trauma, toxins, infection, neurotransmitter abnormalities (in particular in serotonergic or dopaminergic pathways), and autoimmunity. A genetic component has also been suggested, and clues to pathogenesis may be gleaned from the effectiveness or otherwise of pharmacological interventions.

The purpose of this review is to examine the published literature to define what may be inferred with regard to KLS aetiology and to specifically address what evidence there is for an autoimmune aetiology, as first proposed by Dauvilliers [2]. This is of particular interest given the recognition that narcolepsy is most probably an autoimmune disease and the recent growth of immunopsychiatry as a discipline.

Clinical features

The clinical features of KLS are clearly defined [1, 3–8] (Table 1), allowing a secure clinical diagnosis to be made. Most cases are sporadic, although familial cases have been described that seem to present with the same clinical picture as sporadic cases [9]. The diagnostic features of KLS are periodic hypersomnolence, behavioural disorders (in particular eating disturbances and hypersexuality), altered perception, and cognitive dysfunction, with complete clinical recovery between disease episodes [1, 3, 10]. Most patients exhibit also apathy and derealisation [4]. Women appear to experience a longer disease course, lower frequency of hypersexuality, and higher frequency of depressed mood than men [3, 11]. As discussed below, these features suggest that specific areas of the brain may be affected in KLS but do not suggest a specific pathological process. Physical examination is consistently normal, with no focal neurological deficits and no evidence of meningitis [reviewed in 3].

**Table 1:** Diagnostic criteria for Kleine-Levin syndrome as defined by the American Academy for Sleep Medicine [2]

Diagnostic criteria for Kleine-Levin syndrome
Criteria A to E must be met
A. The patient experiences at least two recurrent episodes of excessive sleepiness and sleep duration, each persisting for two days to five weeks.
B. Episodes recur usually more than once a year and at least once every 18 months.
C. The patient has normal alertness, cognitive function, behavior, and mood between episodes.
D. The patient must demonstrate at least one of the following during episodes:
1. Cognitive dysfunction.
2. Altered perception.
3. Eating disorder (anorexia or hyperphagia).
4. Disinhibited behavior (such as hypersexuality).
E. The hypersomnolence and related symptoms are not better explained by another sleep disorder, other medical, neurologic, or psychiatric disorder (especially bipolar disorder), or use of drugs or medications.

Additional clinical features of note when considering the aetiology of KLS are the median age of onset of 15 years (4–82 years, 81% during second decade), male preponderance, infectious prodrome, relapsing-remitting nature of the illness with complete clinical recovery between episodes, ultimate resolution of attacks in most cases, possible HLA associations, and ethnic differences.

Aetiological considerations

Psychological

Psychodynamic theories could explain the behavioural disturbances that, alongside hypersomnolence, characterise KLS [12–14]. Katz and Roper [15] specifically addressed this when describing twins with KLS, believing psychiatric causes to be unlikely. Other authors similarly concluded that psychological theories did not adequately explain the features of KLS. The recent recognition that some behavioural disturbances and psychiatric illnesses may have an underlying autoimmune aetiology is also of interest [16–18].

Neurophysiological and neurochemical abnormalities

Focal epilepsy would be compatible with the relapsing-remitting course of the disease and possible traumatic or infectious triggers. However, there is a consistent lack of electroencephalographic (EEG) evidence of epileptiform activity in patients with KLS [3], although 70% of patients show non-specific diffuse slowing of background EEG activity or, less frequently, low-frequency high-amplitude delta or theta waves mainly in the temporal or temporofrontal areas.

A number of abnormal sleep patterns have been described in KLS [19, 20], but polysomnographic studies have most often been normal [21–23]. A reduction in slow wave sleep early in attacks with a return to normal despite persistent symptoms and, conversely, normal rapid eye movement (REM) sleep early in an attack but decreased REM sleep as the attack progresses, have been described [24].

Examination of biochemical abnormalities in KLS has been unrewarding. Orexin (hypocretin) is of particular interest given the recognition that over 90% of patients with narcolepsy have reduced cerebrospinal fluid (CSF) orexin concentrations [25, 26]. A role for decreased orexin production in KLS has been suggested [27–31]. However, CSF orexin was normal in the patients described by Katz [15] and decreased in only 2 of 7 patients for whom data were available in a review of 186 patients by Arnulf [3]. Lopez [29] reported two patients with decreased CSF orexin levels, one to the levels seen in narcolepsy-cataplexy, but the other with a significant decrease during a disease episode that however remained in the normal range. It is thus suggested that analysis of CSF orexin levels in KLS needs to be studied with care, recognising that more modest episodic reductions may be seen that are not as marked as in narcolepsy-cataplexy [28].

Hypothalamic function tests are of particular interest but results are conflicting, with studies documenting both normal [20, 32–34] and abnormal [35–40] responses in KLS. Hormonal profiles, including melatonin, have been found to be intact, suggesting no circadian disruption [reviewed in 3]. Leptin levels have also been reported as normal in KLS [4]. Methyltetrahdrofolate (MTHF) and neopterin have been reported as mildly decreased or borderline [41].

Catecholamine abnormalities would also be of interest. Occasional patients have shown abnormal serotonin, serotonin metabolite, dopamine, or norepinephrine activity [3, 35, 41, 42], and at post mortem, one patient demonstrated a small locus ceruleus with decreased pigmentation in the substantia nigra [42]. Functional imaging has demonstrated decreased striatal dopamine transporter binding potential in asymptomatic patients with KLS compared to controls [43]. Overall, these findings are not consistent enough to support a pathogenetic hypothesis involving abnormalities of these neurotransmitters. In keeping with this, Dauvilliers [2] found no polymorphisms of catechol-O-methyl transferase or tryptophan hydroxylase genes associated with KLS.

Thus, neurophysiological and biochemical studies exclude epileptiform activity and deficient orexin production of the magnitude seen in narcolepsy as causes of KLS. However, no consistent findings have been described that suggest an alternative aetiology.

Pathology, physiology, and neuroimaging

Multiple interacting neurotransmitter systems in the brain stem, hypothalamus, and basal forebrain converge on common effector systems in the thalamus and cortex to control sleep and wakefulness [44]. The constellation of hypersomnolence, appetite disturbance, and abnormal sexual behaviour suggests hypothalamic dysfunction in particular. However, hypothalamic function seems to be intact in KLS as discussed above. Given the central role of the thalamus in arousal, evidence of thalamic dysfunction is also of particular interest. Guilleminault [45] described two patients with hypersomnia, hypersexuality, and aggressiveness attributed to crack cocaine-induced bilateral vascular lesions of the paramedian nucleus of the thalamus. Similarities between the symptomatology of KLS and that seen in patients with hypothalamic [46] or third ventricle [47] tumours further support the suggestion of diencephalic pathology. In one case report of a patient with KLS, the brain MRI showed decreased diffusion in the splenium of the corpus callosum during the symptomatic phase that resolved completely during the asymptomatic phase [48]. Furthermore, Lu et al. [49] described a patient presenting with apparently classical KLS who had a cystic pineal lesion on MRI. Considering other brain areas that subserve functions relevant to KLS, derealization is an important feature of KLS and is increasingly recognised as a feature that may suggest parieto-temporal dysfunction [50]. Abnormalities in these areas are therefore of particular interest when considering the aetiology of KLS.

Structural lesions are excluded as a cause for KLS by normal CT and MRI findings in all patients that have been studied [3, 51, 52]. Similarly, although a preceding head injury is described in up to 9% of cases, it is not a consistent enough finding for direct brain injury to be the cause in the majority of cases [3, 6, 53]. Finally, structural lesions or direct brain injury do not easily explain the relapsing-remitting nature of disease, particularly as epilepsy can be excluded as discussed above.

Functional imaging studies are of particular interest in defining the anatomical location of brain dysfunction in KLS. Single photon emission tomography (SPECT) [21, 48, 51, 52, 54–57], functional MRI (fMRI) [58, 59], and fluorodeoxyglucose positron-emission tomography (FDG-PET) [60–64] have highlighted multiple brain areas with abnormal perfusion or altered metabolism in KLS (Table 2).

**Table 2:** Summary of functional imaging studies in KLS

	Number of patients	Symptomatic/asymptomatic comparison	Brain areas showing hypoperfusion or functional imaging abnormalities
SPECT			
Hong et al. 2006 [54]	1	Y Subtraction study	Thalamus (R&L), basal ganglia, temporal cortex (L), medial and dorsolateral frontal cortex (R&L), hypothalamus (L) show disease episode specific hypoperfusion
Huang et al. 2005 [51]	7	Symptomatic *n* = 5 Asymptomatic *n* = 7	Thalamus, basal ganglia, temporal cortex, occipital cortex, frontal cortex more marked hypoperfusion when symptomatic. All symptomatic patients restudied when asymptomatic with normalisation of perfusion.
Huang et al. 2012 [21]	30	All patients studied during symptomatic and asymptomatic periods	> 10% reduction when symptomatic in cerebellum, thalamus (L 66.7%, R 11.1%), basal ganglia (L 11.1%, R 22.2%)
Kas 2014 [50]	41	Asymptomatic *n* = 41 Control *n* = 15 Symptomatic *n* = 11	Compared to controls asymptomatic patients had reduced perfusion in the hypothalamus, thalamus, caudate nucleus, and a number of cortical associative areas. When symptomatic patients developed additional hypoperfusion in dorsomedial prefrontal cortex and right parieto-temporal Junction,
Landtblom et al. 2002 [55]	1	Y	Temporal cortex (L>R), frontal cortex (L>R), parietal cortex (R) hypoperfusion. Temporal hypoperfusion persisted when asymptomatic.
Landtblom et al. 2003 [56]	4 (Including patient described in 2002)	Asymptomatic *n* = 4	2/4 Temporal and fronto-temporal hypoperfusion when asymptomatic
Lu et al. 2000 [49]	1	N	Basal ganglia, fronto-temporal cortex, hypothalamus hypoperfusion when symptomatic. Pineal cystic lesion.
Portilla 2002 [57]	1	Y	Left mesiotemporal structures asymptomatic and symptomatic
Vigren et al. 2014 [52]	25	Symptomatic *n* = 16 Asymptomatic *n* = 9	Temporal +/− fronto-temporal and frontal cortex hypoperfusion. No clear difference between symptomatic and asymptomatic studies
MRI/fMRI/Perfusion scintigraphy			
Takayanagi 2017 [48]	1	Y	Symptomatic: brain MRI indicated decreased diffusion in the splenium of the corpus callosum Asymptomatic: the splenial lesion resolved completely
Billings 2011 [65]	1	Y	Reduced perfusion medial thalamus (L). Increased glutamine metabolites thalamus (L) and basal ganglia
Hoexter 2010 [43]	1	Asymptomatic *n* = 1 Controls *n* = 3	TRODAT-1 SPECT: 14% lower striatal DAT in asymptomatic patient compared to controls
FDG-PET			
Dauvilliers 2014 [60]	4	Asymptomatic and symptomatic *n* = 4 Controls *n* = 15	Symptomatic: higher metabolism in paracentral, precentral and postcentral areas, supplementary motor area, medial frontal gyrus, thalamus, and putamen and decreased metabolism in occipital and temporal gyri. Asymptomatic: higher metabolism in frontal and temporal cortices, posterior cingulate and precuneus compared to healthy controls.
Haba-Rubio 2012 [62]	2	Symptomatic *n* = 2 (One receiving lithium) Asymptomatic *n* = 2	Images analysed as averaged images subtracted between symptomatic and asymptomatic periods. Average decrease in metabolic activity in symptomatic periods in hypothalamus, orbitofrontal and frontal parasagittal areas and bilateral posterior regions and a decrease in activity in anterior caudate nuclei, the cingulate and premotor cortex
Lo 2012 [61]	1	Y	Reduced in symptomatic phase in thalamus (R&L), hypothalamus (L), caudate nuclei (R&L), striatum (R&L)
Xie 2016 [63]	1	Y	Symptomatic: hypometabolism in the thalamus and hypothalamus. Mild reduction in cortex. Thalamic/hypothalamic reduction normalised when asymptomatic.
Drouet 2017 [64]	1	Y	Bilateral activation in thalami, caudate nuclei, and lenticular nuclei during symptomatic phase

*SPECT*, single photon emission computerised tomography; *fMRI*, functional magnetic resonance imaging; *FDG-PET*, fluorodeoxyglucose-photon emission tomography

The most consistent finding in SPECT studies is thalamic hypoperfusion during disease episodes [21, 51, 54, 55]. Engström [58] demonstrated thalamic hyperactivation in KLS patients during a working memory test by fMRI. Decreased glucose metabolism has also been observed in the thalamus of symptomatic patients [61, 63]. Magnetic resonance spectroscopy has demonstrated metabolic changes in the thalamus specific to disease episodes [66]. A further study demonstrated a reduction in left medial thalamic perfusion during a disease episode concomitant with an increase in left thalamic glutamine metabolites [65]. A similar increase in glutamine metabolites was seen in the basal ganglia.

Correlation of functional imaging abnormalities with objective assessment of behavioural abnormalities and cognitive function is of particular interest. The Depersonalization/Derealization Inventory Score has been shown to correlate with bilateral hypoperfusion of the parieto-temporal junction during symptomatic periods [50]. The same authors suggested that apathy, seen in KLS, may be caused by right medial prefrontal cortex and orbitofrontal cortex hypoperfusion. In this context, it is of interest that lesions of the orbitofrontal cortex may result also in aberrant eating behaviours [67]. Furthermore, the more the patient suffered from derealization during episodes, the more the bilateral associative posterior cortex (especially the right parieto-temporal cortex) remained affected after the episode [50]. Control subjects did not show the abnormalities demonstrated in asymptomatic KLS patients, suggesting that KLS may not be truly episodic but instead be a persistent (often sub-clinical) abnormality with acute exacerbations. In keeping with this, a child had previously been reported to have left mesiotemporal hypoperfusion that persisted one month after clinical remission [57]. Detailed psychometric testing has demonstrated that memory defects may persist between disease episodes [55, 56, 58, 68] associated with persistent left temporal lobe hypoperfusion.

Kas [50] reported persistent hypothalamic, posterior thalamic, and caudate nucleus hypoperfusion as well as persistent hypoperfusion of the associative cortex in asymptomatic patients compared to controls. This did not correlate with clinical hypersomnolence, which by definition resolved in patients during asymptomatic periods. In contrast, Huang [51] observed the thalamic hypoperfusion demonstrated during disease episodes resolved completely in asymptomatic patients. Finally, Hong [54] demonstrated the diencephalic but also the medial, dorsolateral, and left temporal lobe abnormalities to be more pronounced during symptomatic periods. There may thus be a disparity between diencephalic and some cortical (in particular associative cortex) abnormalities, but further studies are required.

Metabolic studies have produced conflicting results. As above, and in keeping with the suggestion of at least partially reversible diencephalic changes during disease episodes, Xie [63] reported hypothalamic and thalamic hypometabolism, demonstrated by FDG-PET/CT, to reverse significantly during asymptomatic periods. However, Haba-Rubio [62] demonstrated both reduced and increased glucose consumption concomitantly in different brain areas, and Dauvilliers [60] demonstrated widespread hypermetabolism in symptomatic individuals, including in the thalamus, but with hypometabolism in the occipital and temporal gyri. Drouet [64] demonstrated bilateral activation in the thalami, caudate nuclei, and lenticular nuclei during symptomatic periods, consistent with the thalamostriatal system’s regulation of wakefulness and sleep.

In general, these findings support the suggestion that, although remission of the classic diagnostic symptoms is typical of KLS, at least some aspects of the underlying disease process, or the physiological abnormality predisposing to KLS, persist between clinically evident episodes.

It is important to consider what might cause the demonstrated hypoperfusion and metabolic abnormalities. Focal inflammation would be an adequate explanation; inflammatory autoimmune diseases, for example, often following a relapsing-remitting course. A median age of onset of 15 years (but with a very wide range) would also be compatible with this. Huang [51] recognised that inflammation, possibly driven by autoimmune disease, might cause the hypoperfusion they observed on SPECT. Inflammation in the thalamus [69], diencephalon, and mid-brain [70] and abnormalities of the hypothalamus, amygdala, and temporal lobe grey matter [71] have been described. However, histology is available for very few individuals with KLS, and no definitive direct evidence is available to support this hypothesis.

The different brain areas identified by functional neuroimaging and the lack of clarity with regard to the resolution of abnormalities during asymptomatic periods make it difficult to incorporate these findings into a definitive aetiological hypothesis. Further studies with comparison of symptomatic and asymptomatic periods, including where possible healthy controls, and studies that correlate findings of neuroimaging with detailed neuropsychometric testing will hopefully further our understanding of this important area.

Infection and KLS

A number of authors have suggested viral infection as a trigger for KLS [21, 69, 70, 72, 73]. In 168 primary and 18 secondary cases of sporadic KLS reviewed by Arnulf [3], preceding infection was documented in 38.1% of patients who subsequently developed KLS. Reported infections included a flu-like illness, non-specific fever, upper respiratory tract infection and tonsillitis, or ‘summer gastro-enteritis’. In most cases, the infectious agent was not identified, but those that were identified were diverse including EBV, VZV, Asian influenza virus, entero-virus, typhoid vaccine, *Streptococcus*, and scarlet fever.

Kleine-Levin syndrome following acute viral encephalitis has been described [68], and some authors have suggested KLS to be due to focal encephalitis. However, CSF examination was normal in 70 of the cases reviewed by Arnulf [3], and plasma C-reactive protein concentrations have been reported as normal in KLS [4]. A comprehensive study of serum inflammatory cytokines did not reveal any changes during KLS episodes compared to between episodes [73]. Thus, primary CNS infection is unlikely as a cause of KLS. However, infection may trigger autoimmune disease either through cross-reactivity or by providing an inflammatory trigger for autoreactive T cell activation as discussed below.

Autoimmunity

The young age of onset, relapsing-remitting nature of symptoms, infectious prodrome described in many patients, and possible HLA associations are compatible with an autoimmune aetiology. The reported Jewish predisposition and occasional familial cases may also be relevant as they support an inherited component that may predispose to autoimmune disease. It is of interest that a range of sleep disorders have now been associated with auto-antibodies of different specificities causing focal limbic encephalitis or hypothalamic dysfunction [74]. A similar aetiology is possible for KLS.

Immunology of autoimmune disease

Understanding the immunobiology of autoimmune disease explains the inconsistent findings with regard to HLA disease associations and possible infectious triggers. Helper T lymphocytes (Th cells) co-ordinate cellular and humoral (antibody-mediated) immune responses and are thus central to the development of autoimmune disease. They are activated by binding of their T cell receptor (TCR) to a peptide antigen, derived from a target protein, complexed with a class II major histocompatibility complex (MHC) molecule. Human leukocyte antigen (HLA) molecules are the equivalent human MHC molecules.

The development of autoimmunity requires a self-peptide specific to the target tissue to bind a class II HLA molecule and be presented to autoreactive Th cells in a context that allows these Th cells to be activated and manifest their autoreactive phenotype. HLA-binding of peptides is specific, with the amino acid side chains of the peptide interacting with the side chains of the amino acids of the HLA molecule that form the peptide-binding groove. Different HLA molecules have different binding specificity based on the amino acid sequence of their peptide-binding groove. An individual’s HLA phenotype thus determines what peptide antigens will be ‘selected’ and presented to T cells, with some HLA molecules being more likely to bind, and therefore present, self-peptides from a particular tissue to autoreactive T cells. The specific details of these interactions during tolerance induction and antigen presentation in the mature immune system are central to an individual’s susceptibility to autoimmune disease as conferred by the expression of a given HLA molecule [75].

Importantly, many potentially autoreactive T cells are present in the adult immune system that are never activated to cause autoimmune disease. There are two main reasons for this.

First, multiple polymorphic genes influence autoimmune disease susceptibility including genes that determine the background TCR repertoire, genes affecting antigen processing (peptide generation), inherited factors influencing the anti-inflammatory/pro-inflammatory balance of cytokine production during immune responses, genes involved in T cell activation, and a number of genes of no known immune function [76]. Thus, specific HLA haplotypes predispose to autoimmune disease, but these additional factors critically influence whether autoimmune disease is seen.

Second, a trigger is required for T cells to activate and manifest their autoreactive potential. Infection and inflammation resulting from traumatic tissue injury are established triggers.

HLA associations in KLS

Dauvilliers first clearly espoused an autoimmune aetiology for KLS [2], reporting that 16 of 23 patients had an infectious illness preceding KLS onset (13 being a URTI) and 28.3% of KLS patients but only 12.5% of controls expressing the DQB1*0201 allele (*p* ≤ 0.03). Furthermore, Katz [15] described a brother and sister expressing HLA-DR2 and DQ1, who also had flu-like symptoms prior to disease onset. Increased frequencies of HLA-DR1, DQ1 [77], HLA-DQB1*0201 [2], HLA-DQB1*02 [78], HLA-DQB1*0501 [79], HLA-DQB1, DQB1/0602 [21], and HLA-DR2 and DQ1 [15] have all been reported. However, other studies have shown inconsistent HLA associations [80, 81], and larger studies suggest the reported HLA associations may be random [4, 10, 82]. No significant difference is seen in HLA distribution between familial and sporadic cases [83].

Although defining an HLA association is a well-trodden path in the demonstration of an autoimmune aetiology, this inability to define a clear HLA disease association does not preclude an autoimmune aetiology for KLS. First, the MHC is only one autoimmune disease susceptibility factor, this autoimmune potential being critically influenced by multiple additional polymorphic loci. Furthermore, in animal models of neurological autoimmune disease (e.g. experimental autoimmune encephalomyelitis [EAE], an animal model of multiple sclerosis in which the target is myelin), a range of tissue-specific antigens may be the target of autoimmune attack (e.g. myelin basic protein, proteolipoprotein, myelin oligodendroglial glycoprotein) and any given antigen may generate a number of different peptide epitopes capable of causing disease. Each of these autoantigenic epitopes may be presented preferentially by a different MHC molecule. The close association of HLA-DR2 with narcolepsy is therefore the exception rather than the rule. For most autoimmune diseases, multiple permissive peptide/HLA combinations exist, additional susceptibility genes influence disease development, and a trigger is required for the autoimmune potential to manifest itself.

PAMPs and DAMPs as triggers for autoimmune disease

Infection is often considered a trigger for autoimmune disease. This may be due to an infectious agent triggering an immune response that cross-reacts with a self-antigen (e.g. in rheumatic heart disease), but very few autoimmune diseases fit so clearly into such a hypothesis. Infection has also been suggested to cause autoimmune disease by releasing antigens from tissues that are not usually available to the immune system. This may be due to ‘physical’ release or due to inflammation modifying the antigen processing pathways such that ‘new’ peptides are produced (cryptic epitopes) that can cause disease.

Much more likely is the danger hypothesis, first proposed by Matzinger [84]. According to this hypothesis, infection results in an inflammatory signal (pathogen-associated molecular pattern [PAMP]) that lowers the threshold for T cell activation, largely through actions on dendritic cells. This activates autoreactive T cells to cause disease. Molecular mechanisms for these danger signals are now defined [85]. In keeping with this, many animal models of autoimmune disease (e.g. EAE) break tolerance by administering self-antigen in ‘adjuvant’, which induces an intense inflammatory response by including extracts from, for example, mycobacterial strains. However, in keeping with the clinical picture of autoimmune disease, such protocols only induce disease in animal strains that have ‘permissive’ genotypes. Non-infectious injury results in similar non-infectious danger signals (damage-associated molecular patterns—DAMPs), which may also trigger autoimmune disease [86]. A rare but definitive illustration of this is anti-GBM disease following lithotripsy for renal calculi [87].

In general, therefore, an association needs to be shown with a particular agent or group of agents that express cross-reactive antigenic epitopes or with an agent causing infection (and therefore inflammation) in the target organ/tissue. The link with infection as a trigger for autoimmune disease and the complex influence of the genetic background are thus clear. Furthermore, this hypothesis can be extended to other mechanisms of tissue damage that may be relevant to the descriptions of head injury preceding KLS onset.

Clues from therapeutic interventions

It is of interest to consider what pharmacological interventions are of benefit in KLS. Stimulants such as modafinil may have benefit [88] but may mask symptoms rather than truly modifying the underlying disease process [4]. Risperidone may improve psychotic symptoms [4] and normalise sleep cycles [89]. Dopamine agonists, if used early [4, 8, 10], and gabapentin [89] may both be effective. Carbamazepine [90, 91], lamotrigine [92], valproate [4, 93, 94], and lithium [4, 95–99] have all been reported to be beneficial, and interestingly these medications are also reported to have anti-inflammatory properties [100–104]. Perhaps surprisingly given the putative autoimmune aetiology, steroids have infrequently been used, with a recent case report of an influenza A-triggered case of KLS being treated with dexamethasone on initial presentation with uncertain benefit [48].

In an open-label controlled study of 130 patients, lithium decreased the frequency and duration of KLS attacks [105]. A recent case report described long-term (2 years) episode-free disease management with low-dose lithium [106]. Arnulf [3] reported that only lithium has response rates (prevention of relapse) greater than no medical intervention. In keeping with this, a recent Cochrane Review found only lithium to have significant evidence of benefit, but concluded that no evidence was available to support any specific therapy in KLS due to the lack of adequately constructed randomised controlled trials [107]. The need for double-blind, placebo-controlled, randomised, controlled trials of drugs to prevent or improve KLS symptoms, although challenging, is clear. The immunopathology of KLS suggests that drugs that modulate or target the immune system are worthy of further testing in clinical trials [108].

## Summary

The diagnostic clinical features of KLS may be explained by the specific neuroanatomical location of cerebral dysfunction. It is also important to determine the cause of this dysfunction. Importantly, more than one process may be responsible in different individuals, akin to Parkinson’s disease, where similar clinical features may be caused by infection, vascular insult, or most often degenerative disease.

This review reaches the inescapable conclusion that the available evidence best fits with an autoimmune aetiology, as first proposed by Dauvilliers [2]. It is difficult to directly confirm this, in particular as brain histology is not available and no animal model exists. A co-ordinated international effort to consistently diagnose, investigate, and manage KLS is progressing our understanding of this disease. Genetic studies examining the many polymorphisms affecting immune responsiveness [73] would be of interest. Ultimately, the possibility of an autoimmune aetiology will only be addressed by a co-ordinated multi-centre randomised interventional study using immunosuppressive drugs, which should now be possible. Although individual case reports have suggested IVIg treatment to be of benefit in narcolepsy [109], overall results have been disappointing [71]. These findings should sound a note of caution and emphasise the importance of careful choice of immunosuppressive strategy in clinical trial design.

Practice points

1. Clinical diagnosis should be made with reference to the AASD ICSD-3 (2014) criteria. The reviews of Arnulf and Billiard are also of particular use.

2. Clinical examination, electrophysiological studies, and CNS imaging are expected to be normal in KLS patients.

3. The therapeutic intervention most likely to be of benefit is lithium, although proof of efficacy is lacking.

4. Functional neuroimaging may be helpful but is not diagnostic. Referral to a sleep centre with expertise in diagnosing and managing Kleine-Levin syndrome is recommended.

Research agenda

1. The recent Cochrane Review of treatment was inconclusive, largely due to small numbers of informative patients. A co-ordinated multi-centre, double-blind, randomised, controlled trial of treatment would be of benefit.

2. An autoimmune aetiology is suggested, but not confirmed, by available data. This could be address further by:

Genetic analysis of loci associated with autoimmune disease

Therapeutic trials of immunosuppressive agents

3. Functional imaging studies comparing symptomatic and asymptomatic periods and including healthy controls is of interest, in particular to define persistent abnormalities in asymptomatic patients compared to healthy controls.

## Abbreviations

KLS: Kleine-Levin syndrome
HLA: Human leukocyte antigen
MHC: Major histocompatibility complex
CT: Computerised tomography
MRI: Magnetic resonance imaging
fMRI: Functional MRI
SPECT: Single photon emission tomography
FDG-PET: Fluorodeoxyglucose-Photon emission tomography
TCR: T cell receptor
Th: Helper T cell
EAE: Experimental autoimmune encephalomyelitis
PAMP: Pathogen-associated molecular patterns
DAMP: Damage-associated molecular patterns
EEG: Electroencephalogram
REM: Rapid eye movement
CSF: Cerebrospinal fluid

## References

[1] American Academy of Sleep Medicine. International Classification of Sleep Disorders - Third Edition (ICSD-3). (2014) Darien (IL): American Academy of Sleep Medicine

[2] Dauvilliers Y, Mayer G, Lecendreux M, Neidhart E, Peraita-Adrados R, Sonka K, Billiard M, Tafti M (2002) Kleine-Levin syndrome: an autoimmune hypothesis based on clinical and genetic analyses. Neurology 59(11):1739–45. 10.1212/01.WNL.0000036605.89977.D0 10.1212/01.wnl.0000036605.89977.d0 12473762

[3] Arnulf I, Zeitzer JM, File J, Farber N, Mignot E (2005) Kleine–Levin syndrome: a systematic review of 186 cases in the literature. Brain 128(12):2763–76, 10.1093/brain/awh620 10.1093/brain/awh620 16230322

[4] Arnulf I, Lin L, Gadoth N, File J, Lecendreux M, Franco P, Zeitzer J, Lo B, Faraco JH, Mignot E (2008). Kleine-Levin syndrome: a systematic study of 108 patients. Ann Neurol.

[5] Arnulf I, Rico TJ, Mignot E (2012) Diagnosis, disease course, and management of patients with Kleine-Levin syndrome. Lancet Neurol 11(10):918–928. 10.1016/S1474-4422(12)70187-4 10.1016/S1474-4422(12)70187-4 22995695

[6] Billiard M, Jaussent I, Dauvilliers Y, Besset A (2011) Recurrent hypersomnia: a review of 339 cases. Sleep Med Rev 15(4):247–57. 10.1016/j.smrv.2010.08.001 10.1016/j.smrv.2010.08.001 20970360

[7] Billiard M, Peraita-Adrados R, Tafti M, Shaw P, Tafti M, Thorpy M (2013). Genetics of recurrent hypersomnia. The genetic basis of sleep and sleep disorders.

[8] Miglis MG, Guilleminault C (2014). Kleine-Levin syndrome: a review. Nat Sci Sleep.

[9] Al Suwayri SM (2016). Kleine-Levin syndrome. Familial cases and comparison with sporadic cases. Saudi Med J.

[10] Arnulf I (2015). Kleine-Levin syndrome. Sleep Med Clin.

[11] Nebhinani N, Suthar N (2017). Sleeping beauty syndrome: a case report and review of female cases reported from India. Indian J Psychol Med.

[12] Markman RA (1967). Kleine–Levin syndrome: report of a case. Am J Psychiatry.

[13] Masi G, Favilla L, Millepiedi S (2000). The Kleine–Levin syndrome as a neuropsychiatric disorder: a case report. Psychiatry.

[14] Miller DL (1970). Kleine–Levin syndrome: an atypical case?. Psychiatr Q.

[15] Katz JD, Ropper AH (2002). Familial Kleine-Levin syndrome: two siblings with unusually long hypersomnic spells. Arch Neurol.

[16] Ching KH, Burbeloa PD, Carlson PJ, Drevets WC, Ladarolaa MJ (2010). High levels of anti-GAD65 and anti-Ro52 autoantibodies in a patient with major depressive disorder showing psychomotor disturbance. J Neuroimmunol.

[17] Kruse JL, Lapid MI, Lennon VA, Klein CJ, Toole OO, Pittock SJ, Strand EA, Frye MA, McKeon A (2015). Psychiatric autoimmunity: N-methyl-D-aspartate receptor IgG and beyond. Psychosomatics.

[18] Padmos RC, Bekris L, Knijff EM, Tiemeier H, Kupka RW, Cohen D, Nolen WA, Lernmark Å, Drexhage HA (2004). A high prevalence of organ-specific autoimmunity in patients with bipolar disorder. Biol Psychiatry.

[19] Gadoth N, Kesler A, Vainstein G, Peled R, Lavie P (2001). Clinical and polysomnographic characteristics of 34 patients with Kleine-Levin syndrome. J Sleep Res.

[20] Mayer G, Leonhard E, Krieg J, Meier-Ewert K (1998). Endocrinological and polysomnographic findings in Kleine-Levin syndrome: no evidence for hypothalamic and circadian dysfunction. Sleep.

[21] Huang YS, Guilleminault C, Lin KL, Hwang FM, Liu FY, Kung YP (2012). Relationship between Kleine-Levin syndrome and upper respiratory infection in Taiwan. Sleep.

[22] Luo YW, Yu H, Yuan LH, Zhu GX (2016). A polysomnography study of Kleine-Levin syndrome in a single center. Chin Med J.

[23] Reimao R, Shimizu MH (1998). Kleine-Levin syndrome. Clinical course, polysomnography and multiple sleep latency test. Case report Arquivos de Neuro-Psiquiatria.

[24] Huang YS, Lin YH, Guilleminault C (2009). Polysomnography in Kleine-Levin syndrome. Neurology.

[25] Bourgin P, Zeitzer JM, Mignot E (2008). CSF hypocretin-1 assessment in sleep and neurological disorders. Lancet Neurol.

[26] Liblau RS, Vassalli A, Seifinejad A, Tafti M (2015). Hypocretin (orexin) biology and the pathophysiology of narcolepsy with cataplexy. Lancet Neurol.

[27] Cho JW, Kim JH (2016). Recurrent hypersomnia in an 18-year old boy: CSF hypocretin level and MSLT findings. Sleep Med.

[28] Dauvilliers Y, Lopez R (2016). Time to find a biomarker in Kleine-Levin syndrome. Sleep Med.

[29] Lopez R, Barateau L, Chenini S, Dauvilliers Y (2015). Preliminary results on CSF biomarkers for hypothalamic dysfunction in Kleine-Levin syndrome. Sleep Med.

[30] Podesta C, Ferreras M, Mozzi M, Bassetti C, Dauvilliers Y, Billiard M (2006). Kleine-Levin syndrome in a 14-year-old girl: CSF hypocretin-1 measurements. Sleep Med.

[31] Wang JY, Han F, Dong SX, Li J, An P, Zhang XZ, Chang Y, Zhao L, Zhang XL, Liu YN, Yan H, Li QH, Hu Y, Lv CJ, Gao ZC, Strohl KP (2016). Cerebrospinal fluid orexin A levels and autonomic function in Kleine-Levin syndrome. Sleep.

[32] Hart EJ (1985). Kleine-Levin syndrome normal CSF monoamines and response to lithium therapy. Neurology.

[33] Hasegawa Y, Morishita M, Suzumura A (1998). Novel chromosomal abberation in a patient with a unique sleep disorder. J Neurol Neurosurg Psychiatry.

[34] Thompson C, Obrecht R, Franey C, Arendt J, Checkley S (1985). Neuroendocrine rhythms in a patient with the Kleine-Levin syndrome. Br J Psychiatry.

[35] Chesson AL, Levine SN, Kong LS, Lee SC (1991). Neuroendocrine evaluation in Kleine-Levin syndrome: evidence of reduced dopaminergic tone during periods of hypersomnolence. Sleep.

[36] Fernández JM, Lara I, Gila L, O’Neill A, Tovar J, Gimeno A (1990). Disturbed hypothalamic-pituitary axis in idiopathic recurring hypersomnia syndrome. Acta Neurol Scand.

[37] Fukunishi I, Hosokawa K (1989). A female case with the Kleine-Levin syndrome and its physiopathologic aspects. Psychiatry Clin Neurosci.

[38] Gadoth N, Dickerman Z, Bechar M, Laron Z, Lavie P (1987). Episodic hormone secretion during sleep in Kleine-Levin syndrome: evidence for hypothalamic dysfunction. Brain and Development.

[39] Livrea P, Puca F, Barnaba A, Di Reda L (1977). Abnormal central monoamine metabolism in humans with ‘true hypersomnia’ and ‘sub-wakefulness’. Eur Neurol.

[40] Malhotra S, Das MK, Gupta N, Muralidharan R (1997). A clinical study of Kleine-Levin syndrome with evidence for hypothalamic pituitary axis dysfunction. Biol Psychiatry.

[41] Maranci JB, Roze E, Benoist JF, Mochel F, Rigal O, Arnulf I (2017). Dopamine and serotonin levels in cerebrospinal fluid during episodes of Kleine-Levin syndrome. Sleep Med.

[42] Koerber RK, Torkelson R, Haven G, Donaldson J, Cohen SM, Case M (1984). Increased cerebrospinal fluid 5-hydroxytryptamine and 5-hydroxyindoleacetic acid in Kleine-Levin syndrome. Neurology.

[43] Hoexter MQ, Shih MC, Mendes DD, Godeiro-Junior C, Felicio AC, YK F (2008). Lower dopamine transporter density in an asymptomatic patient with Kleine-Levin syndrome. Acta Neurol Scand.

[44] Brown RE, Basheer R, McKenna JT, Strecker RE, McCarley RW (2012). Control of sleep and wakefulness. Physiol Rev.

[45] Guilleminault C, Querra-Salva MA, Goldberg MP (1993). Pseudo-hypersomnia and pre-sleep behaviour with bilateral paramedian thalamic lesions. Brain.

[46] Haugh RM, Markesbery WR (1983). Hypothalamic astrocytoma. Syndrome of hyperphagia, obesity, and disturbances of behavior and endocrine and autonomic function. Arch Neurol.

[47] Fulton JF, Bailey P (1929). Tumors in the region of the third ventricle: their diagnosis and relation to pathological sleep. J Nerv Ment Dis.

[48] Takayanagi M, Okabe S, Yamamoto K, Komatsu J, Suzuki R, Kitamura T, Ohura T (2017). Kleine–Levin syndrome elicited by encephalopathy with reversible splenial lesion. Pediatrics Int.

[49] ML L, Liu HC, Chen CH, Sung SM (2000). Kleine–Levin syndrome and psychosis: observation from an unusual case. Neuropsychiatry Neuropsychol Behav Neurol.

[50] Kas A, Lavault S, Habert MO, Arnulf I (2014) Feeling unreal: a functional imaging study in 41 patients with Kleine-Levin syndrome. Brain 1377(7):2077–87. 10.1093/brain/awu112 10.1093/brain/awu112 24785943

[51] Huang YS, Guilleminault C, Kao PF, Liu FY (2005) SPECT findings in the Kleine-Levin syndrome. Sleep 28(8):955–60, 10.1093/sleep/28.8.955 10.1093/sleep/28.8.955 16218078

[52] Vigren P, Engstrom M, Landtblom AM (2014). SPECT in the Kleine-Levin syndrome, a possible diagnostic and prognostic aid?. Front Neurol.

[53] Will RG, Young JPR, Thomas DJ (1988). Report of two cases with onset of symptoms precipitated by head trauma. Br J Psychiatry.

[54] Hong SB, Joo EY, Tae WS, Lee J, Han SJ, Lee HW (2006). Episodic diencephalic hypoperfusion in Kleine-Levin syndrome. Sleep.

[55] Landtblom AM, Dige N, Schwerdt K, Säfström P, Granérus G (2002). A case of Kleine–Levin syndrome examined with SPECT and neuropsychological testing. Acta Neurol Scand.

[56] Landtblom AM, Dige N, Schwerdt K, Säfström P, Granérus G (2003) Short-term memory dysfunction in Kleine–Levin syndrome. Acta Neurol Scand 108(5):363–7. 10.1034/j.1600-0404.2003.00171.x 10.1034/j.1600-0404.2003.00171.x 14616308

[57] Portilla P, Durand E, Chalvon A, Habert M, Navelet Y, Prigent A, Landrieu P (2002). SPECT-identified hypoperfusion of the left temporomesial structures in a Kleine–Levin syndrome. Rev Neurol (Paris).

[58] Engström M, Karlsson T, Landtblom AM (2014). Thalamic activation in the Kleine-Levin syndrome. Sleep.

[59] Engström M, Hallböök T, Szakacs A, Karlsson T, Landtblom AM (2014). Functional magnetic resonance imaging in narcolepsy and the Kleine–Levin syndrome. Front Neurol.

[60] Dauvilliers Y, Bayard S, Lopez R, Comte F, Zanca M, Peigneux P (2014). Widespread hypermetabolism in symptomatic and asymptomatic episodes in Kleine-Levin syndrome. PLoS One.

[61] Lo YC, Chou YH, Yu HY (2012). PET finding in Kleine-Levin syndrome. Sleep Med.

[62] Haba-Rubio J, Prior JO, Guedj E, Tafti M, Heinzer R, Rossetti AO (2012). Kleine-Levin syndrome: functional imaging correlates of hypersomnia and behavioral symptoms. Neurology.

[63] Xie H, Guo J, Liu H, Song W (2016). Do the symptoms of Kleine-Levin syndrome correlate with the hypometabolism of the thalamus on FDG PET?. Clin Nucl Med.

[64] Drouet C, Morel O, Verger A, Guedj E, Boulahdour H (2017). FDG brain PET/CT revealing bilateral thalamostriatal activation during a symptomatic episode in a patient with Kleine-Levin syndrome. Clin Nucl Med.

[65] Billings ME, Watson NF, Keogh BP (2011). Dynamic fMRI changes in Kleine-Levin syndrome. Sleep Med.

[66] Poryazova R, Schnepf B, Boesiger P, Bassetti CL (2007). Magnetic resonance spectroscopy in a patient with Kleine-Levin syndrome. J Neurol.

[67] Butter CM, McDonald JA, Snyder DR (1969). Orality, preference behavior, and reinforcement value of nonfood object in monkeys with orbital frontal lesions. Science.

[68] Fontenelle LF, Mendlowicz MV, Marques C, Mattos P, Versiani M (2003). Persistent neuropsychological deficits in the Kleine-Levin syndrome. Acta Neurol Scand.

[69] Carpenter S, Yassa R, Ochs R (1982). A pathologic basis for Kleine-Levin syndrome. Arch Neurol.

[70] Fenzi F, Simonati A, Crosato F, Ghersini L, Rizzuto N (1993). Clinical features of Kleine-Levin syndrome with localized encephalitis. Neuropediatrics.

[71] Takrani LB, Cronin D (1976). Kleine-Levin syndrome in a female patient. Can Psychiatr Assoc J.

[72] Merriam AE (1986). Kleine-Levin syndrome following acute viral encephalitis. Biol psychiatry (1993) a variant of the Kleine-Levin syndrome precipitated by both Epstein-Barr and varicella-zoster virus infections. Biol Psychiatry.

[73] Kornum BR, Rico T, Lin L, Huang YS, Arnulf I, Jennum P, Mignot E (2015). Serum cytokine levels in Kleine-Levin syndrome. Sleep Med.

[74] Silber MH (2016) Autoimmune sleep disorders handbook of clinical neurology, Vol. 133 (3rd series) pp 317-25 Autoimmune Neurology S.J. Pittock and A. Vincent, Editors Elsevier B.V 10.1016/B978-0-444-63432-0.00018-9 27112685

[75] Liu GY, Fairchild PJ, Smith RM, Prowle JR, Kioussis D, Wraith DC (1995). Low avidity recognition of self-antigen by T cells permits escape from central tolerance. Immunity.

[76] Gregersen PK, Olsson LM (2009) Recent advances in the genetics of autoimmune disease. Ann Rev Immunol 27(1):363–91, 10.1146/annurev.immunol.021908.132653 10.1146/annurev.immunol.021908.132653 PMC2992886 19302045

[77] Manni R, Martinetti M, Ratti MT, Tartara A (1993). Electrophysiological and immunogenetic findings in recurrent monosymptomatic-type hypersomnia: a study of two unrelated Italian cases. Acta Neurol Scand.

[78] BaHammam AS, GadElRab MO, Owais SM, Alswat K, Hamam KD (2008). Clinical characteristics and HLA typing of a family with Kleine-Levin syndrome. Sleep Med.

[79] Rocamora R, Gil-Nagel A, Franch O, Vela-Bueno A (2010). Familial recurrent hypersomnia: two siblings with Kleine-Levin syndrome and menstrual-related hypersomnia. J Child Neurol.

[80] Ueno T, Fukuhara A, Ikegami A, Ohishi F, Kume K (2012). Monozygotic twins concordant for Kleine-Levin syndrome. BMC Neurol.

[81] Visscher F, van der Horst AR, Smit LM (1990). HLA-DR antigens in Kleine-Levin syndrome. Ann Neurol.

[82] Lavault S, Golmard JL, Groos E, Brion A, Dauvilliers Y, Lecendreux M, Franco P, Arnulf I (2015). Kleine-Levin syndrome in 120 patients: differential diagnosis and long episodes. Ann Neurol.

[83] Nguyen QT, Groos E, Leclair-Visonneau L, Monaca-Charley C, Rico T, Farber N (2016). Familial Kleine Levin syndrome: a specific entity?. Sleep.

[84] Matzinger P (1994) Tolerance, danger, and the extended family. Annu Rev Immunol 12(1):991–1045. 10.1146/annurev.iy.12.040194.005015 10.1146/annurev.iy.12.040194.005015 8011301

[85] Bianchi ME (2007). DAMPs PAM: Ps and alarmins: all we need to know about danger. J Leukoc Biol.

[86] Hirsiger S, Simmen H-P, Werner CML, Wanner GA, Rittirsch D (2012). Danger signals activating the immune response after trauma. Mediat Inflamm.

[87] Cranfield A, Mathavakkannan S (2015). Goodpasture’s disease following extracorporeal shock wave lithotripsy: a case report & literature review. Clin Case Rep.

[88] Adenuga O, Attarian H (2014). Treatment of disorders of hypersomnolence. Curr Treat Options Neurol.

[89] Itokawa K, Fukui M, Ninomiya M, Yamamoto T, Imabayashi E, Tamura N, Matsuda H, Araki N (2009). Gabapentin for Kleine-Levin syndrome. Internal Med.

[90] Perin BV, Rodrigues I, Giasson FT, Balen M, Posenato N, Forcelini CM (2015). Recurrent hypersomnia: report of medication-responsive cases. Sleep Sci.

[91] Mukaddes NM, Kora ME, Bilge S (1999). Carbamazepine for Kleine-Levin syndrome. J Am Acad Child Adolesc Psychiatry.

[92] Surges R, Walker MC (2009). A case of late-onset Kleine-Levin syndrome responding to lamotrigine. Sleep Med.

[93] Adlakha A, Chokroverty S (2009). An adult onset patient with Kleine-Levin syndrome responding to valproate. Sleep Med.

[94] Crumley FE (1997). Valproic acid for Kleine-Levin syndrome. J Am Acad Child Adolesc Psychiatry.

[95] Das S, Gupta R, Dhyani M, Raghuvanshi S (2014). Kleine-Levin syndrome: a case report and review of literature. Pediatr Neurol.

[96] Goldberg MA (1983). The treatment of Kleine-Levin syndrome with lithium. Canadian J Psychiatry.

[97] Mendhekar DN, Jiloha RC, Gupta D (2001). Kleine-levin syndrome: a report of two cases. Indian J Psychiatry.

[98] Muratori F, Bertini N, Masi G (2002). Efficacy of lithium treatment in Kleine-Levin syndrome. European Psychiatry.

[99] Poppe M, Friebel D, Reuner U, Todt H, Koch R, Heubner G (2003). The Kleine-Levin syndrome—effects of treatment with lithium. Neuropediatrics.

[100] Bazinet RP, Rao JS, Chang L, Rapoport SI, Lee HJ (2006). Chronic carbamazepine decreases the incorporation rate and turnover of arachidonic acid but not docosahexaenoic acid in brain phospholipids of the unanesthetized rat: relevance to bipolar disorder. Biol Psychiatry.

[101] Lee HJ, Ertley RN, Rapoport SI, Bazinet RP, Rao JS (2008). Chronic administration of lamotrigine downregulates COX-2 mRNA and protein in rat frontal cortex. Neurochem Res.

[102] Chang MC, Contreras MA, Rosenberger TA, Rintala JJ, Bell JM, Rapoport SI (2001). Chronic valproate treatment decreases the in vivo turnover of arachidonic acid in brain phospholipids: a possible common effect of mood stabilizers. J Neurochem.

[103] Rapoport SI, Bosetti F (2002). Do lithium and anticonvulsants target the brain arachidonic acid cascade in bipolar disorder?. Arch Gen Psychiatry.

[104] Nassar A, Azab AN (2014). Effects of lithium on inflammation. ACS Chem Neurosci.

[105] Leu-Semenescu S, Le Corvec T, Groos E, Lavault S, Golmard JL, Arnulf I (2015). Lithium therapy in Kleine-Levin syndrome: an open-label, controlled study in 130 patients. Neurology.

[106] Parmar A, Yadav P, Patra BN, Sagar R (2017). Successful long-term management of a child with Kleine-Levin syndrome with low-dose lithium. Indian J Psychol Med.

[107] de Oliveira MM, Conti C, Prado GF (2016) Pharmacological treatment for Kleine-Levin syndrome. Cochrane Database Syst Rev (5):Cd006685 10.1002/14651858.CD006685.pub4 PMC7386458 27153153

[108] Al Suwayri SM, BaHammam AS (2017). The “known unknowns” of Kleine-Levin syndrome: a review and future prospects. Sleep Med Clin.

[109] Dauvilliers Y, Abril B, Mas E, Michel F, Tafti M (2009). Normalization of hypocretin-1 in narcolepsy after intravenous immunoglobulin treatment. Neurology.

